# Dual-Resolution Dual-Path Convolutional Neural Networks for Fast Object Detection

**DOI:** 10.3390/s19143111

**Published:** 2019-07-14

**Authors:** Jing Pan, Hanqing Sun, Zhanjie Song, Jungong Han

**Affiliations:** 1 School of Electrical and Information Engineering, Tianjin University, Tianjin 300072, China; 2 School of Mathematics, Tianjin University, Tianjin 300072, China; 3 WMG Data Science, University of Warwick, Conventry CV4 7AL, UK

**Keywords:** dual-resolution, CNN, visual object detection, progressive fusion

## Abstract

Downsampling input images is a simple trick to speed up visual object-detection algorithms, especially on robotic vision and applied mobile vision systems. However, this trick comes with a significant decline in accuracy. In this paper, dual-resolution dual-path Convolutional Neural Networks (CNNs), named DualNets, are proposed to bump up the accuracy of those detection applications. In contrast to previous methods that simply downsample the input images, DualNets explicitly take dual inputs in different resolutions and extract complementary visual features from these using dual CNN paths. The two paths in a DualNet are a backbone path and an auxiliary path that accepts larger inputs and then rapidly downsamples them to relatively small feature maps. With the help of the carefully designed auxiliary CNN paths in DualNets, auxiliary features are extracted from the larger input with controllable computation. Auxiliary features are then fused with the backbone features using a proposed progressive residual fusion strategy to enrich feature representation.This architecture, as the feature extractor, is further integrated with the Single Shot Detector (SSD) to accomplish latency-sensitive visual object-detection tasks. We evaluate the resulting detection pipeline on Pascal VOC and MS COCO benchmarks. Results show that the proposed DualNets can raise the accuracy of those CNN detection applications that are sensitive to computation payloads.

## 1. Introduction

In robotic applications, there is a trend of integrating robotics with human beings and their environments. Reliable object detection in real-time speed is usually a necessary early step to achieve interaction between robots and environments. However, object detection is a challenging task in robotic vision, and Convolutional Neural Network (CNN)-based object-detection methods have emerged in the mainstream [1,2,3,4,5,6] due to their great performance in complex scenes. Among them, Faster R-CNNs [6,7,8], Feature Pyramid Network (FPN) variants [1,9], YOLO [5], and Single Shot Detectors (SSD) [2] are some of the mainstream approaches that have shown to be accurate and/or efficient. Despite the immense success of those methods, the high computational cost of large-scale CNN models still hinders applications of CNN-based detectors on embedded systems, such as mobile phones, self-driving cars, and drones.

A widely used trick to lower the computational cost of a CNN model is to downsample input images to lower resolutions. This trick, despite instant results, is known to sacrifice detection accuracy [2,10,11]. Because computation becomes squared if one merely doubles the input width, it is not feasible to accept large inputs on all applied systems.

To address this problem, efficient CNN models were proposed for embedded devices and have achieved high inference speed with a non-negligible accuracy drop [10,11,12]. For example, depthwise separable convolutions were proposed in MobileNets [10] to bring down the computational cost. These mobile-oriented CNN architectures, as feature extractors, are used in conjunction with the detectors mentioned above, resulting in CNN-based object-detection pipelines with high inference speed but limited detection accuracy. By integrating SSD [2] and MobileNets, the resulting MobileNet–SSD pipeline achieved the state-of-the-art performance of mobile models on visual object detection [11]. As a trade-off between computational cost and accuracy, the MobileNet–SSD pipeline with depthwise separable convolutions achieved high inference speed but limited accuracy.

**Figure 1:**
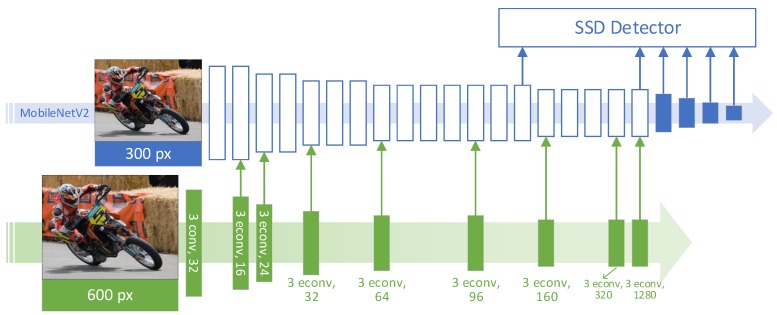
Architecture of proposed DualNets. There are dual paths in a DualNet: upper path is the backbone convolutional neural network (CNN) architecture (e.g., MobileNetV2 [11]); the lower is auxiliary path with larger inputs but less stacked layers. Feature maps in the auxiliary path are fused with the corresponding backbone feature in a residual-learning manner as long as their dimensions meet.

To diminish existing performance gaps, in this paper we propose DualNets, dual-resolution dual-path CNNs, to bump up the accuracy of object-detection applications that are sensitive to computation payloads such as those deployed on embedded devices. DualNets are designed to extract features from dual inputs in different resolutions aiming to enrich visual features. To be specific, the dual paths in a DualNet consist of a backbone path (MobileNetV2 in Figure 1) and an auxiliary path, which accepts larger inputs (e.g., 600 pixels squared, which is twice the input width of the backbone path) and then rapidly downsamples them to relatively small feature maps. A feature map from the auxiliary path is fused with the corresponding backbone feature as long as their dimensions meet; this strategy is named progressive fusion in this paper. We further developed a novel residual-learning formula as the core of the progressive fusion strategy.

**Figure 2:**
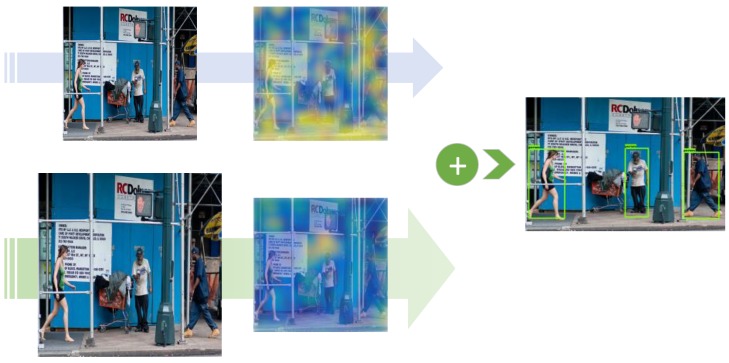
How visual features are enriched in DualNets. Heatmaps show complementary features extracted from small (**top**) and large (**bottom**) inputs. According to residual-learning theory, the magnitudes of the auxiliary features are relatively small. Largest magnitude values (clipped to a maximum of 5) are mapped yellow, while the smallest are mapped dark blue.

Figure 2 shows the motivation of the proposed fusion strategy in this paper. The two CNN paths in Figure 2 are the backbone path (top) and the auxiliary path (bottom), and heatmaps are the two-norm of feature maps extracted from the example image from the MS COCO dataset [13]. Backbone features are extracted from small inputs by the backbone path. Auxiliary features are extracted from the large inputs by the designed auxiliary path, which has fewer stacked layers to reduce computational costs. Fusing those complementary features from both paths with a progressive fusion strategy helps to improve the detection results. Experiment results show that the designed fusion strategy, which is based on a novel form of residual learning, contributes to an overall accuracy gain. Spatial resolutions of the feature maps in the auxiliary paths are kept low using rapidly downsampling CNN streamlines to keep computational costs affordable on embedded devices. Figure 1 is an illustration of the proposed DualNets architecture.

We summarize our contributions as follows:

(1) A novel dual-resolution dual-path framework, DualNets, was designed to enhance CNN-based object-detection applications that are sensitive to computational payloads. DualNets consist of dual CNN paths taking different input resolutions and holding complementary features, resulting in strengthened capability for visual-feature representation.
(2) The auxiliary paths in DualNets were designed to accept larger inputs to enrich visual features for object detection. The auxiliary feature maps were then rapidly downsampled to lower overall computation payloads. With such design patterns, computational cost can be flexibly controlled.
(3) Feature maps extracted by auxiliary paths are progressively fused into the backbone CNN streamline. We developed a novel form of residual learning [14], which is the core of the proposed progressive fusion strategy. Applying the fusion strategy on complementary features extracted by the dual paths, DualNets can raise the accuracy of mobile-oriented CNN detectors.

## 2. Related Work

In this section, we briefly introduce related works in three aspects. Several representative visual object-detection methods are first reviewed. Then, state-of-the-art CNN architectures that focus on mobile or embedded platforms are described. Third, we compare our DualNets with other methods that also hold dual CNN paths.

### 2.1. CNN-Based Object Detection

Current mainstream CNN-based detectors can be divided into two categories: anchor-based detectors and anchorfree detectors. It is noted that anchor-based detectors can be categorized as two-stage detectors [6] and single-stage detectors [2]. An anchor [6] is a predefined reference box (i.e., the default box) centered at a given position in a feature map, and is associated with the scale and aspect ratio. Anchor-based detectors then predict offsets that are parameterized relative to *k* anchors, where *k* denotes the number of anchors (k=9 in this paper).

A faster R-CNN [6] is one of the forerunners of the two-stage detectors. This widely used detection framework consists of a CNN-based feature extractor, a region proposal network, and a classifier. The two stages can then be defined as the proposal generation stage (i.e., the body) and the proposal recognition stage (i.e., the head) [15], and there is a large number of detectors following this framework. FPN [1] is aimed at detecting multiscale objects by upsampling the feature maps of the CNN feature extractor, resulting in considerable performance gains in small- and medium-scale object detection. Because higher-resolution feature maps are used for smaller-scale object detection, many FPN variants enrich those features in various approaches [9,16,17].

In contrast with the two-stage methods, unified CNN pipelines are exploited in single-stage detectors [2,5,18,19,20]. YOLO [5] and SSD [2] are two representative one-stage detectors. In YOLO [5], a single neural network is used to directly predict proposals and class probabilities from input images. Another unified CNN pipeline was designed in SSD [2] in order to meet the challenges of multiscale detection, where multiresolution feature maps are taken into account. In SSD, those multiresolution feature maps are directly extracted from backbone CNN features instead of upsampling the smaller feature maps, as done in FPN.

To meet real-time demands on embedded devices, single-stage detectors such as SSD [2], are preferred over two-stage ones. Therefore, we integrated SSD into the proposed DualNets and conducted the experiments with such integration in this paper.

### 2.2. Fast Inference Using Small CNN Models

Inference efficiency is one of the primary considerations on embedded object-detection systems.

Multilayer Channel Features (MCF) [21] is an object-detection framework that can reject numbers of irrelevant regions early to avoid further computation. Sharing a similar motivation to MCF, another early-prediction solution, GlanceNets [22], was proposed to reduce average inference time. Pointwise group convolutions and channel shuffle operations, which can both reduce computation cost with limited accuracy drop, were proposed in ShuffleNet [12]. Depthwise separable convolutions were proposed in MobileNets [10,11] to bring down computational costs.

By integrating MobileNets with SSD [2], object detection can be conducted on modern mobile phones. As a trade-off between computational cost and accuracy, the MobileNet–SSD pipeline achieved promising inference speed but limited accuracy.

Downsampling inputs of those models to lower resolutions (e.g., 300 pixels) can further reduce computation to a square-root level while suffering from a significant drop in accuracy. To solve the dilemma, we introduce a novel architecture that can extract features from dual-resolution inputs without the squared computational costs.

### 2.3. Dual-Path Models

In this subsection, existing representative dual-path networks are briefly reviewed. We then distinguish the proposed DualNets with those dual-path CNN models.

The first family of dual-path CNN architectures is knowledge distillation [23,24], which usually consists of a teacher network and a student network. It is introduced to accelerate and compress the CNN models [23] and has many variants [24,25,26,27]. Following [23], distillation is exploited on object-detection tasks with a mimicking network [24]. Those distillation methods are aimed at training a more compact model that can learn from the output of a large model, that is, the teacher network (the larger model) is used to only help the student network (the smaller model) at the training stage. The teacher network is then trimmed off at the inference stage. The main drawback of knowledge distillation is that the teacher network is large not only in feature map resolutions but also in the depth of the stacked CNN layers, resulting in complicated training stages. It is worth noting that there is no hindrance to prevent imposing model mimicking or distilling on DualNets.

A Guided Upsampling Module [28] was recently proposed to guide the decoder layers within an encoder-decoder semantic-segmentation pipeline using an extra-large-input weight-sharing CNN path. In contrast with a Guided Upsampling Network (GUN) [28] for semantic segmentation, proposed DualNets are armed at object detection on embedded devices. The extra path output is only fused into the decoder using multilayer fusion modules in the GUN, whereas auxiliary feature maps are progressively fused into the backbone in the proposed DualNets. Moreover, the avoidance of weight sharing gives us the flexibility to design a rapidly downsampling auxiliary path that achieves less computation but has strengthened representation capability.

In this paper, aiming to improve the detection accuracy of small CNN models, enriched features were extracted by the novel auxiliary residual path where high-resolution input images were rapidly downsampled. With the designed progressive-fusion strategy, auxiliary features were fused with the backbone; thus, result features were enhanced.

## 3. DualNets: Dual-Input Dual-Path CNNs

The details of the proposed DualNets are described in this section. Some preliminary approaches are briefly reviewed in Section 3.1, as DualNets was designed mainly based on MobileNetV2–SSD [10,11]. The composition of DualNets, including dual inputs, dual paths, and progressive-fusion strategy, is then introduced in Section 3.2 and Section 3.3.

### 3.1. Brief Review of MobileNets and SSD

DualNets are composed of depthwise separable convolutions proposed in MobileNets [10] and SSD [2]. We give a brief review of those cornerstones in this subsection.

A depthwise separable convolution [10] is a form of convolutions that is factorized into a depthwise convolution and a pointwise (1×1) convolution. In a depthwise convolution, only a single convolution filter is applied in each channel, in contrast with multiple filters used in a classic convolution. Computation can be drastically reduced with the help of depthwise separable convolutions, resulting in fast inference on embedded vision systems. Depthwise separable convolutions are widely used [10,11,12] and are building blocks of the proposed DualNets.

**Figure 3:**
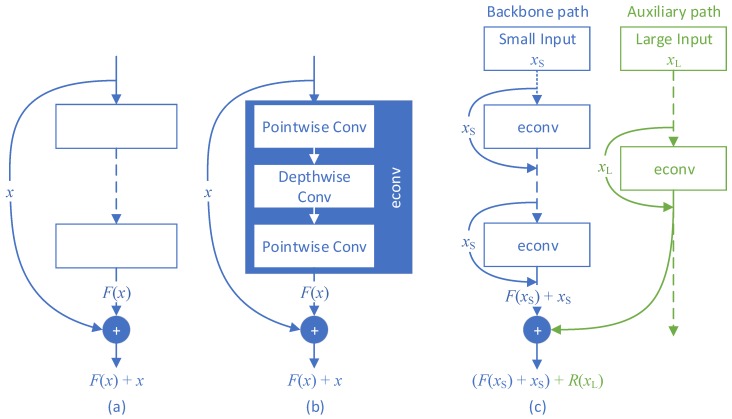
Different forms of residual learning. (**a**) Classical skip-connection form of residual learning; (**b**) inverted residual block used in MobileNetV2 [11]; (**c**) proposed residual learning formula.

Bottleneck-with-expansion layers, denoted as Expanded depthwise separable CONVolution (econv) layers in this paper for simplicity, were proposed upon the depthwise separable convolution layers in MobileNetV2 [11]. Figure 3b is an illustration of an econv layer. The input feature map of an econv layer is first expanded in channels by pointwise convolution and then fed into depthwise convolution, followed by another pointwise convolution to project the channel-expanded feature map to the desired channel size. MobileNetV2 [11] is a stack of one 3×3 convolutional layer, seventeen 3×3 econv layers (2nd–18th layers), and one 1×1 convolutional layer (the 19th layer), as shown with the hollow blue boxes in Figure 1. MobileNetV2 can be integrated with SSD [2] as the feature extractor to enable the result MobileNetV2–SSD pipeline to detect objects on mobile devices. Four pairs of one-stride 1×1 and two-stride 3×3 convolutional layers were cascaded after the MobileNetV2 architecture (solid blue boxes in Figure 1) to extract lower-resolution features for larger-scale object detection. Output feature maps of the expansion convolution of the 15th econv layer, the 19th layer, and the last four layer pairs are fed into the SSD (blue arrows in Figure 1). By doing so, feature maps at six distinct resolutions are integrated for multiscale object detection.

Despite the multiscale detection capability and fast inference of MobileNetV2–SSD, detection accuracy is still limited. We propose DualNets to improve the detection accuracy of small detection models, and we chose the representative mobile-oriented MobileNet–SSD pipeline as a baseline. The dual paths in the proposed framework were designed according to a novel form of residual learning that could help to extract enriched features with affordable computation.

### 3.2. Dual Inputs and Dual Paths

It is natural to use high-resolution inputs to help low-resolution models with a dual-input or multi-input design as done in [28,29]. We argue that merely stacking input images into image pyramids is not applicable to all vision systems in consideration of the squared computational payloads. Therefore, a lightweight auxiliary path that has controllable computational costs is introduced in DualNets (colored in green in Figure 1).

A DualNet accepts two inputs, a lower-resolution image and a higher-resolution one. The former is directly fed into the backbone MobileNetV2 feature extractor, and the latter is fed into the designed auxiliary path to help enrich the extracted features. In the auxiliary path, inputs are rapidly downsampled with strided convolutions in the first several layers. To be specific, we stacked one 3×3 convolutional layer and eight 3×3 econv layers (Layers 2–9), resulting in a lightweight CNN path with nine layers in total. As shown by the solid green boxes in Figure 1, the first four layers (Layers 1–5) are convolutions with stride 2. Thus, the output resolutions of those rapid downsampling layers are, respectively, 300, 150, 75, 38, and 19 pixels. Following the design pattern of MobileNetV2, the sixth econv layer is responsible for transiting feature depths (the channel dimensions) instead of downsampling. The feature maps are further downsampled into 10 pixels by the seventh econv layer, which is followed by two nonstrided econv layers for feature-depth transition. A detailed comparison of MobileNetV2 and DualNets is shown in Table 1, where key configuration parameters are presented side by side.

By utilizing computational-analysis tools of econvs [10], the computation costs of each convolution in an econv layer can be calculated as:

(1) $$C_{\mathrm{point}1}=M\cdot \left(M\cdot t\right)\cdot D_{F}^{2},$$

(2) $$C_{\mathrm{depth}}=D_{k}^{2}\cdot \left(M\cdot t\right)\cdot \frac{D_{F}^{2}}{s^{2}},$$

(3) $$C_{\mathrm{point}2}=\left(M\cdot t\right)\cdot N\cdot \frac{D_{F}^{2}}{s^{2}},$$

where *M* denotes the number of input channels, *N* denotes the number of output channels, *t* denotes the expansion ratio as described in [11], *s* is the stride of the econv layer, $D_{k}^{2}\;\left(=D_{k}\cdot D_{k}\right)$ denotes the kernel size of the depthwise separable convolution, and $D_{F}^{2}\;\left(=D_{F}\cdot D_{F}\right)$ denotes the resolution of the input feature map. There are expansion pointwise convolution ($C_{\mathrm{point}1}$), depthwise separable convolution ($C_{\mathrm{depth}}$), and projection pointwise convolution ($C_{\mathrm{point}2}$) in an econv layer. Thus, the computational cost of the econv layer is:

(4) $$\begin{array}{cc} C_{\mathrm{econv}} & =C_{\mathrm{point}1}+C_{\mathrm{depth}}+C_{\mathrm{point}2}\\ & =\left(M+\frac{D_{k}^{2}+N}{s^{2}}\right)\cdot \left(M\cdot t\right)\cdot D_{F}^{2}. \end{array}$$

According to Equation (4) and Table 1, the total computational cost of MobileNetV2–SSD with a 300-pixel input is around 854 M Multiply-Adds, and the number becomes 3416 M when using MobileNetV2 on 600-pixel inputs, whereas it is 688 M Multiply-Adds for the auxiliary path in DualNets. It is also notable that configurations of auxiliary paths in DualNets can be changed to meet various requirements. Shown in this paper is an experimental configuration to illustrate the proposed DualNets framework and progressive residual fusion strategy, which is introduced in the following subsection.

It is noted that Batch Normalization (BN) and the ReLU activation function were employed after the convolutional layers.

### 3.3. Progressive Residual Fusion

Residual learning was introduced into CNNs in ResNet [14] aiming to address the degradation problem in training deep CNN models. The hypothesis of residual learning is that fitting residual mappings of features is easier than learning the original mappings. Shortcut connection (as shown in Figure 3a) is the original and widely used implementation of residual learning. It is also used in MobileNetV2 [11] in the form of the inverted residual block, where the inputs of an econv are added to the outputs (as shown in Figure 3b). In this paper, we introduce a novel residual-learning approach that was designed for DualNets as well as any other model that requires fusion from two asymmetric pipelines.

The proposed residual-learning formula is different from the classical in that appended residual mappings can be captured from the higher-resolution inputs by the auxiliary path in DualNets. Figure 3c is an illustration of the proposed residual-learning approach. Classical residual learning implemented with shortcut connection (Figure 3a) can be denoted as:

(5) H(x)=F(x)+x,

where *x* is the input of a single-input CNN model, such as ResNets [14,30]. As forcing the model to fit desired mapping H(x) suffers from degradation [14], residual mapping F(x) is learned instead, which is substantially easier to fit than desired mapping H(x). Thus the desired mapping becomes the summation of identical mapping *x* and residual mapping F(x), as shown in Equation (5). In the proposed DualNets (Figure 3c), on the other hand, the formula of desired mapping H(x) is extended as:

(6) $$H\left(x\right)=\left(F\left(x_{S}\right)+x_{S}\right)+R\left(x_{L}\right),$$

where $x_{S}$ denotes the original small inputs of DualNets, and $x_{L}$ denotes the large auxiliary inputs. First term $F\left(x_{S}\right)+x_{S}$ is the desired mapping whose inputs are the smaller images. Existing methods using this mapping for detection suffer from limited accuracy because all details are blurred during downsampling. An extra term, residual mapping fitted from large inputs $R(x_{L})$, is therefore introduced into the new residual-learning equation. The design philosophy of $R(x_{L})$ lies on two facts: (1) Simply enlarging input width causes squared increment in model computation; (2) Dual paths in DualNets are asymmetric, that is, they do not share similar layer stacks. Expecting a simple model as the auxiliary path to fit the desired mapping results in underfitting that harms detection. We leveraged the computational cost and model capability with the novel form of residual learning. The auxiliary path plays the role of $R(x_{L})$, and Equation (6) was implemented by the proposed progressive residual fusion in DualNets.

The remaining problem is where the formula should be performed. The most intuitive fusion strategy is to fuse feature maps from dual paths after the feature-extraction workflow, that is, the dual paths work independently and are fused together before detection. Experiment results (described in Section 4.1) show that such a simple design pattern cannot bring performance gains. Thus, we designed a progressive-fusion strategy. As the name suggests, fusions take place progressively when information flows in asymmetric DualNets, resulting in enhanced feature extraction and representation.

As shown in Table 1, there can be multiple layers holding the same output channel size in a CNN model. For example, there are four layers (Layers 8–11) that hold 64-channel feature maps in the backbone path. Thus, auxiliary feature maps sharing the same channel size (the fifth auxiliary layer) can be fused into any of the four backbone layers. We then explored two progressive-fusion strategies: early and late fusion. In the early progressive-fusion strategy, output feature maps in the auxiliary path are fused with the backbone as long as their output dimensions meet. Retake the above example: the 64-channel output from the fifth auxiliary layer is fused with the output of the eightth backbone layer according to the early fusion strategy. The summation of those two then becomes the input of the ninth backbone layer. In the late progressive-fusion strategy, the output feature maps in the auxiliary path are fused with the last outputs within the sequence of layers with matching dimensions. For the 64-channel example, the fifth auxiliary feature maps are fused with the output of the 11th backbone layer.

The difference between the two progressive-fusion strategies is that, from the perspective of the proposed form of residual learning, fusion results are further utilized as inputs of subsequent layers in early fusion, which makes the following residual mapping ($F(x_{S})$ in Equation (6)) much easier to fit. Because small models, such as MobileNets, suffer more from underfitting rather than overfitting [10], making the mapping easy-to-fit benefits the training of DualNets. Comparison experiments were conducted on the proposed progressive residual fusion, and results are shown in Section 4.1.

Because the input of the upper path of the DualNet described in Figure 1 and Table 1 is of resolution of 300×300 pixels, we denoted it DualNet-300. Similarly, we could construct DualNet-512 where the resolution of the input of the upper path is 512×512 pixels. The architecture of DualNet-512 is the same as that of DualNet-300 except for two differences: (1) input resolution of the upper path of DualNet-512 is 512×512 pixels; (2) the first layer of the auxiliary path of DualNet-512 is resized to 256×256 pixels by bilinear interpolation after stride-2 convolution with 3×3 kernels.

## 4. Experiments

The DualNet-300 configuration for experiments is as shown in Table 1 and Figure 1.

The difference between DualNet-512 and DualNet-300 is described at the end of Section 3. Results are compared with those from our trained MobileNetV2–SSD [2,11] model.

Ablation studies of DualNet-300 (Section 4.1) were conducted on the Pascal VOC dataset [31]: Training and validation data from both Pascal VOC 2007 and 2012 (named trainval0712) were used for training, and the labeled test data from Pascal VOC 2007 (named test2007) were used for validation. The DualNet model for the MS COCO dataset [13] (Section 4.2) was trained with the trainval35k dataset [6] and was evaluated using the test-dev2018 dataset by the MS COCO evaluation sever. Results in this section were measured by the mean Average Precision (mAP), mAP for medium-scale objects, and mAP for large-scale objects from the MS COCO dataset, which are more comprehensive. The metrics for small-scale objects were omitted because it is too challenging to detect them using downsampled input images by mobile-oriented models. The mAP numbers for Pascal VOC were measured by mAP at 0.5 mean Intersection over Union (mIoU) for comparison, which is one of the original Pascal VOC metrics.

### 4.1. Ablation Study on Dualnet-300

**Figure 4:**
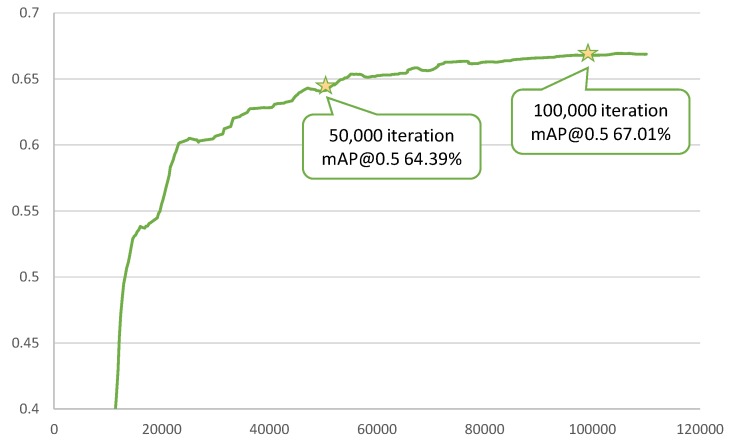
Validation mAP curves at mIOU threshold 0.5: a comparison between 50 k and 100 k iteration training. According to our observation, models trained around 50,000 iterations are sufficient for comparison in ablation studies. Thus, results in Section 4.1 are reported as those of 50 k-iteration trainings.

Ablation experiments in this subsection were conducted to analyze the impact of weight sharing, initialization, and fusion strategies. Taking publicly available MobileNetV2 checkpoint pretrained on the ILSVRC classification dataset [32,33], we finetuned the experimental DualNet model (Table 1) using SGD with an initial learning rate of $2\times 10^{-3}$ and a batch size of 24. The learning rate was lowered by a factor of 0.7 every 10,000 iterations. Other hyperparameters were set following MobileNets [10,11]. Aiming at demonstrating the roles of the proposed components in DualNets, most of the training in this subsection was stopped at near 50,000 iterations, which is acceptable for an ablation study that is not yet fully converged. Curves of validation accuracy (during training) on the Pascal VOC test2007 dataset are shown in Figure 4 as an illustration of the difference between 50 k and 100 k iteration training.

#### 4.1.1. Weight Sharing

The GUN [28] mentioned in Section 2.3 is a CNN model that accepts dual inputs as DualNets do. GUN differs from our proposed DualNets in that two paths in the GUN share the same structures and weights. Considering that dual paths in DualNets are asymmetrical, both in input resolutions and structures, we argue that forcing those two paths to share weights is harmful.

According to the configurations of the dual paths (shown in Figure 1 and Table 1), the depths of those are distinct. For example, the 15th layer in the backbone has an identical form of weights to that of the seventh layer in the auxiliary; thus, their weights can be shared. As stacking more layers helps with the model capability of feature representation, the capability gap between a 15th layer and a seventh cannot be ignored for such a small model. To test that hypothesis, experiments on DualNets with weight sharing were performed. Specifically, weights of all layer pairs shown in the same row in Table 1 were shared, e.g., weights of the 15th backbone layer and the seventh auxiliary layer were shared. Results (Table 2) show that forcefully sharing layer weights is harmful when those layers are at different levels, i.e., when they have significant capability gaps.

#### 4.1.2. Initializing from a Pretrained Model

Another intuitive approach to initialize weights in the auxiliary path is to load them from a pretrained MobileNetV2 model. Initialization from the MobileNetV2 weights pretrained on the ILSVRC dataset [32,33] can provide a good starting point for the auxiliary path during model optimization. However, as mentioned above, existing gaps between backbone layers and auxiliaries caused by the asymmetry cannot be diminished. To explore the impact of this initialization method, layer pairs as defined in weight-sharing experiments were initialized using the pretrained MobileNetV2 model. Initialized identically, those weights were then independently finetuned, in contrast with the aforementioned weight-sharing strategy. The results (denoted as Pretrained in Table 2), compared with the results of weight-sharing experiments, show that the pretrained model does provide a good start.

However, rethinking Equations (5) and (6) in residual learning [14,30] (Section 3.3), our appended residual mappings $R(x_{L})$ were expected to be easy-to-fit mapping, especially for small models such as MobileNets and DualNets. Thus, a random initialized auxiliary path, along with a pretrained MobileNetV2 model initialized backbone path was trained according to our proposed residual-learning formula (Equation (6)). Results of that exploration are shown in the Random row in Table 2. It is shown that random initialization on the auxiliary path outperformed the other two strategies, which further demonstrates that the proposed form of residual learning can diminish level gaps caused by layer asymmetry.

#### 4.1.3. Fusion Strategy

Following the flow in Section 3, the remaining problems are fusion timing and method.

Concatenation and summation are two widely used fusion methods in CNN models [14,28,29,30,34]. Thus, comparison experiments between concatenation and summation were conducted on the experimental configuration of DualNet. The architecture shown in Figure 1 and Table 1 remained unchanged, except that outputs of a layer pair were concatenated in the concatenation experiments (denoted as concat. in Table 3) instead of being element-wise summed in the summation experiments (denoted as res. in Table 3, as summation is the default in the classical residual-learning context [14,30]).

For fusion timing, experiments were conducted to compare fusion before detection, progressive late fusion, and progressive early fusion. The first strategy was to only fuse (concatenate or sum) features from the last three auxiliary layers with the backbone features from Layers 15, 18, and 19, respectively. In our proposed progressive-fusion strategy, fusions were performed as long as the feature-map dimensions met. To be specific, in the progressive early fusion strategy, outputs were fused together and results were fed into the subsequent backbone layer for each layer pair in Table 1. As described in Section 3.3, auxiliary features were fused with the backbone features before the next strided convolutional layer in the progressive late fusion strategy. Results shown in Table 3 demonstrate that the proposed progressive residual fusion strategy helps with both medium-scale and larger-scale object detection. The designed progressive early fusion outperformed other strategies, so we used this strategy for the final experimental model.

### 4.2. Results

We trained the DualNet-300 with theconfiguration of Table 1 and Figure 1 using the progressive early fusion strategy.

The DualNet-512 was trained in the same manner. Note that the chosen learning rates were lowered tens of times faster than in MobileNets [10] and only a single GPU was used during training due to resource limitation, resulting in a performance drop on the metrics compared with that reported by MobileNetV2 [11]. Aiming to introduce a way of incorporating high-resolution inputs with controllable computational cost, we reported the experiment results trained on our own for a fair comparison.

For the Pascal VOC dataset, most training parameters were the same as used in Section 4.1, except for the model that was trained for around 100,000 iterations. Results in terms of the detection mAP and detection time are given in Table 4. Compared with the MobileNetV2–SSD detection pipeline, the proposed DualNet-300 could achieve mAP 67.01% with mIoU threshold 0.5 and have higher accuracy on all metrics (see Table 4). Moreover, both MobileNetV2–SSD and DualNet spent much less time for inference.

For the challenging MS COCO dataset, the initial learning rate was set to $2\times 10^{-3}$ and was lowered by a factor of 0.7 every 20,000 iterations. Both the baseline MobileNetV2–SSD and the proposed DualNets were trained on our own with batch size 24 on a single NVIDIA 1080 Ti GPU for fair comparison. Results are given in Table 5. The results demonstrate that both the proposed DualNet-300 and DualNet-512 with dual paths fused by progressive fusion outperformed the corresponding baselines of MobileNetV2-SSD-300 and MobileNetV2-SSD-512, especially on the mAP for large-scale objects.

**Figure 5:**
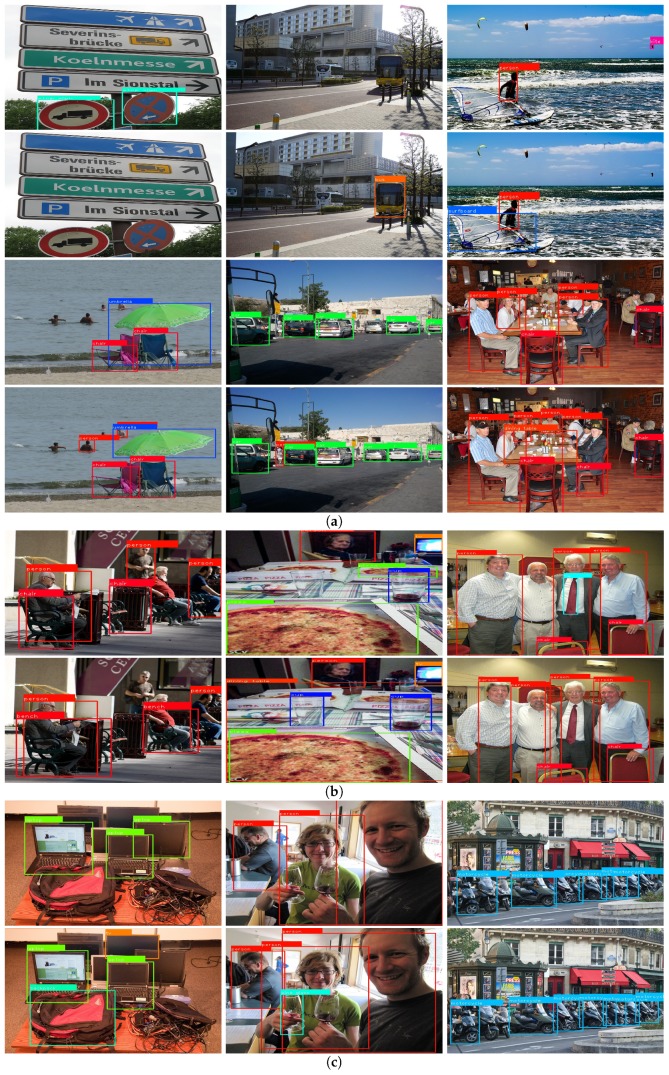
Representative detection results. (**a**) More foreground objects could be detected by the DualNet-300 model. (**b**) Some cases in which DualNet-300 performs not as well as the MobileNetV2–SSD. (**c**) Some challenging examples for both MobileNetV2–SSD-300 and DualNet-300.

Figure 5 visualizes detection results. For every two rows in Figure 5, the upper shows the results of MobileNetV2–SSD-300 and the lower shows those of the proposed DualNet-300. Figure 5a shows that more foreground objects could be detected by the DualNet-300 model. For example, in the first row, the nearer bus in the second image and the surfboard in the third could be detected using our DualNet. Figure 5b presents some cases in which DualNet-300 performs not as well as the MobileNetV2–SSD. According to our observation, DualNets have better performance on large-scale objects that cover a large portion of pixels in an image (e.g., the cup in the second image of Figure 5b), which is a common design purpose in some embedded vision systems. Figure 5c is some challenging examples for both MobileNetV2–SSD-300 and DualNet-300. The two detectors could not manage to detect the ambiguous, occluded, or truncated objects in those images. Despite the performance gain brought by the designed DualNets, tackling those problems in embedded systems remains a challenge. It is concluded from Figure 5 that the proposed DualNets is capable of reducing false negatives because the proposed auxiliary path introduces detailed and discriminative information to the upper path (backbone).

## 5. Conclusions

We presented a CNN framework, DualNets, that accepts dual inputs in different resolutions and consists of dual paths that hold asymmetric CNN models. Our method is aimed at extracting enriched visual features from higher-resolution input images without suffering from squared computational cost. A fusion strategy called progressive fusion based on a novel form of residual learning was designed to diminish the capacity gaps between two asymmetric CNN models in DualNets. While we only presented an exemplary configuration of our DualNets on the MobileNetV2–SSD pipeline, this approach could also be flexibly applied to other small detection models that are sensitive to computation payloads. In future work, we will apply our object detector to facilitate real-world applications, including image retrieval [35,36], image tracking [37], and image classification [38].

## Figures and Tables

**Table 1:** Detailed comparison of MobileNetV2 and DualNets. Layers denoted in form of operation types and kernel sizes in the op. ker. column; corresponding strides and channels shown in the str. and the ch. columns.

DualNets							
MobileNetV2							
#	op. ker.	str.	ch.	#	op. ker.	str.	ch.
input 300px		-	3	input 600px		-	3
1	conv.3	2	32	1	conv.3	2	32
2	econv.3	1	16	2	econv.3	2	16
3	econv.3	2	24	3	econv.3	2	24
4	econv.3	1	24				
5	econv.3	2	32	4	econv.3	2	32
6	econv.3	1	32				
7	econv.3	1	32				
8	econv.3	2	64	5	econv.3	2	64
9	econv.3	1	64				
10	econv.3	1	64				
11	econv.3	1	64				
12	econv.3	1	96	6	econv.3	1	96
13	econv.3	1	96				
14	econv.3	1	96				
15	econv.3	2	160	7	econv.3	2	160
16	econv.3	1	160				
17	econv.3	1	160				
18	econv.3	1	320	8	econv.3	1	320
19	conv.1	1	1280	9	econv.3	1	1280

**Table 2:** Comparison of auxiliary-path initialization strategies. For the weight-sharing entry, weights were shared between auxiliary path and backbone. Pretrained means the auxiliary path was initialized with the pretrained model but independently finetuned. The auxiliary was randomly initialized in the Random entry. The Pascal VOC trainval0712 dataset was used for training, and test2007 for the test in the ablation-study experiments (Section 4.1).

Initializer	mAP	mAP (Medium Scale)	mAP (Large Scale)
Weight-sharing	50.07%	7.88%	36.20%
Pre-trained	63.43%	10.93%	45.72%
Random	64.39%	10.93%	46.45%

**Table 3:** Comparison of fusion timing and methods. In the timing column, det. stands for fusion only before detection (i.e., only the last three auxiliary layers were fused into the backbone); prog. late stands for progressive late fusion, and prog. early is our proposed form of progressive residual fusion. For the fusion methods, concat. stands for fused using concatenation; res. denotes the form of residual learning.

Timing	Method	mAP	mAP (Medium Scale)	mAP (Large Scale)
Det.	concat.	58.51%	9.51%	41.32%
Det.	res.	59.39%	9.56%	42.35%
Prog. late	res.	61.45%	9.99%	45.44%
Prog. early	res.	64.39%	10.93%	46.45%

**Table 4:** Results of DualNet-300 compared with MobileNetV2–SSD-300 [11] on Pascal VOC dataset.

	mAP@IoU0.5 100 k iter.	mAP (Medium Scale)	mAP (Large Scale)	Time (ms)
MobileNetV2–SSD-300	66.48%	12.16%	48.91%	6.18
DualNet-300	67.01%	12.45%	49.09%	10.7
Faster R-CNN (VGG, 600 px)	70.40%	–	–	110

**Table 5:** Results of DualNets compared with MobileNetV2–SSD [11] on the MS COCO dataset.

	mAP@IoU.5:.05:.95	mAP (Medium Scale)	mAP (Large Scale)
MobileNetV2–SSD-300	13.7%	10.5%	27.0%
DualNet-300	14.1%	10.8%	28.4%
MobileNetV2–SSD-512	15.2%	12.1%	28.4%
DualNet-512	15.6%	12.5%	29.5%
